# A Cross-Sectional Study of Pre-Prepared Foods Knowledge, Attitudes, and Practices of College Students in Central China

**DOI:** 10.3390/nu16193267

**Published:** 2024-09-27

**Authors:** Reyisaimu Wumaierjiang, Yijia Xu, Lei Wang, Taotao Guo, Guoxun Chen, Rui Li

**Affiliations:** 1 School of Public Health, Wuhan University, Wuhan 430071, China; 2019303050018@whu.edu.cn (R.W.); xuyijia0507@163.com (Y.X.); lleiwang@whu.edu.cn (L.W.); taotaoguo@whu.edu.cn (T.G.); 2 College of Food Science and Technology, Huazhong Agricultural University, Wuhan 430070, China; 3 College of Biomedicine and Health, Huazhong Agricultural University, Wuhan 430070, China; 4 School of Nursing, Wuhan University, Wuhan 430071, China; 5 Research Center for Lifespan Health, Wuhan University, Wuhan 430071, China

**Keywords:** knowledge, attitude, practice, pre-prepared foods, Chinese college students

## Abstract

**Objectives:** This study aimed to investigate knowledge, attitudes, and practices related to pre-prepared foods among college students in Central China. **Methods:** From the end of May 2024 to June 2024, we completed a cross-sectional study using an online questionnaire. A total of 1676 questionnaires were distributed online, and 1566 valid questionnaires were collected. Data were analyzed using Kruskal–Wallis tests or Wilcoxon rank-sum tests for univariate analysis. A multiple linear regression model was employed with knowledge, attitudes, and practices scores as dependent variables to identify factors associated with the scores on pre-prepared food knowledge, attitudes, and practices. **Results:** The survey results showed that 56.7% of the participants had high knowledge scores, 4% of the participants had high attitudes scores, and only 0.4% of the participants had high practices scores. Multiple linear regression analysis showed that ethnicity, the number of children in the family, academic qualifications, and monthly living expenses were associated with college students’ knowledge of pre-prepared foods (*p* < 0.05). Gender and the satisfaction with school catering services were associated with college students’ attitudes of pre-prepared foods (*p* < 0.05). Gender, knowledge and attitudes were associated with practices of pre-prepared foods (*p* < 0.05). **Conclusions:** College students have a relatively high level of knowledge of pre-prepared foods. However, they have more negative attitudes and practices towards pre-prepared foods, and the overall KAP levels are low.

## 1. Introduction

Recently, the popularity of pre-prepared foods, also known as convenient or pre-cooked meals, has increased significantly. This popularity can be attributed to their ability to save time and eliminate the hassle associated with the preparation of traditional meals. Pre-prepared foods encompass a variety of meals that are either partially or fully prepared in advance, requiring minimal cooking or effort before consumption. This category includes items ranging from ready-to-eat meals, frozen dinners, and meal kits to canned and vacuum-sealed products. In China, the popularity of pre-prepared foods has increased in recent years [1]. This expansion may be fueled by several key factors, including rapid urbanization, the fast-paced nature of modern lifestyles, and continuous advancements in food preservation and preparation technologies. Urbanization has led to a rise in the number of people living in cities, where busy schedules and demanding jobs leave little time for cooking. Consequently, many individuals turn to convenient food options that allow them to maintain a balanced diet without the need for extensive cooking [2]. According to statistics from Zhao et al. [3], the Chinese pre-prepared foods market reached CNY 415.15 billion in 2022, with 64,000 pre-prepared foods enterprises currently operating. Sales forecasts suggest that the pre-prepared foods market will continue to grow at an annual rate of approximately 20% over the next three to five years. By 2026, the market is expected to reach CNY 1072 billion annually. This indicates that the pre-prepared foods industry has enormous potential for further development in China [4,5]. However, the consumption of pre-prepared foods has raised health concerns [6]. Studies have shown that these foods often contain high levels of sodium, saturated fats, and preservatives, which are associated with various health issues such as hypertension, obesity, and cardiovascular diseases [7,8,9]. However, little is known about the correlates of pre-prepared food consumption, which might be targeted for intervention.

University students represent a significant and rapidly expanding consumer group for pre-prepared foods. This demographic is particularly receptive to new trends and technologies, making them early adopters of convenience foods [10,11]. The majority of Chinese university students live in dormitories with limited cooking facilities, such as small shared kitchens that are often equipped with only basic appliances like microwaves and electric kettles [12]. Furthermore, these students frequently lack the necessary culinary skills or the time required to prepare meals from scratch due to their rigorous academic schedules and extracurricular commitments [13,14]. This reliance is further driven by the affordability and accessibility of these food options, which align well with the typical student budget and busy lifestyle [15,16]. Consequently, many students rely heavily on take-out, dining hall options, and pre-prepared meals, leading to high exposure to pre-prepared foods [17]. The convenience, affordability, and ease of use make pre-prepared foods an attractive option for students, but it also raises concerns about their nutritional intake and long-term health impacts.

The Knowledge, Attitude, and Practice (KAP) model is a widely used theoretical framework for understanding health-related behaviors [18]. This model examines the interplay between individuals’ knowledge about a topic, their attitudes towards it, and their subsequent practices or behaviors [19,20]. Applying the KAP model to the consumption of pre-prepared foods among university students can provide valuable insights into their dietary habits and the factors influencing these behaviors. Research in this area is crucial for developing targeted interventions to promote healthier eating habits among students.

In summary, this study aims to investigate the current state of knowledge, attitudes, and practices regarding pre-prepared foods among university students. By exploring the factors influencing their consumption patterns, the research seeks to provide evidence-based recommendations to guide students towards healthier and more balanced diets. The findings are expected to contribute to the development of educational and policy initiatives that support the well-being of university students.

## 2. Materials and Methods

### 2.1. Participant Selection

We conducted a cross-sectional survey from the end of May 2024 to June 2024. The participants of this study needed to meet the conditions as follows: 1. university student in Wuhan, China; 2. volunteering to participate in the study; 3. using a smartphone, tablet, computer, or other device to fill out the questionnaire. And the exclusion criteria were as follows: 1. unfinished questionnaire; 2. there are contradictions in what they fill in. For example, there are two types of items, forward scoring and reverse scoring, and there are inconsistencies if all the answers to all items in a questionnaire are the same.

### 2.2. Data Collection and Quality Management

For the present study, a convenience sample of college students was recruited in Central China. Participants were recruited via WeChat Moments, a commonly used social media in China. From the end of May 2024 to June 2024, we shared the recruitment information in a total of 10 WeChat Moments and 20 WeChat groups, and provided a QR code leading to the electronic questionnaire, and they were asked to answer several questions about age and education in progress, major, and grade level, and based on the answers to these questions, were judged whether or not they were college students and whether or not they could be included in our study. We received 1676 responses to the questionnaire. Of those, 110 provided incomplete data and were removed from the sample, leaving 1566 participants for analysis. Ethical and anonymous data use statements were declared at the beginning of the questionnaire and agreed upon by the expectant. After collecting the questionnaires, two rounds of screening were conducted to eliminate invalid responses, including questionnaires with incomplete information and contradictions. The research protocol and design were reviewed, revised, and approved by the Biomedical Ethics Committee of Wuhan University (Approval No.:WHU-LFMD-IRB2024025; 20 May 2024).

### 2.3. Sample Size Calculation

Using Cochran’s formula $n_{0}=Z^{2}pq$/$e^{2},$ the sample size was calculated to be 1067, where $n_{0}$ = Cochran’s sample size recommendation, Z is 1.96 at a 95% confidence interval, e is the margin of error at 3% (standard deviation of 0.03), and q = 1 − p. Since there was no prior research on the knowledge, attitudes, and practices regarding pre-prepared foods among Chinese university students, p = 50% was used. Subsequently, the following modified Cochran’s formula was used for calculating the adjusted sample size in a small population: $n=n_{0}N$/$[N+(n_{0}-1)];$ here, n = adjusted sample size, $n_{0}$ = 1067 (Cochran’s sample size recommendation), and N represented the total population size, which was 47.6319 million in 2023, encompassing all forms of higher education in China. The sample size for this calculation was 1067. This study collected 1566 valid questionnaires, exceeding the calculated sample size.

### 2.4. Questionnaire Design

The researchers designed the questionnaire using the KAP model, utilizing literature review and group discussion methods. The questionnaire comprised 41 questions divided into four sections:

1. Sociodemographic characteristics with 17 items;
2. Knowledge about pre-prepared foods with eight items;
3. Attitudes towards pre-prepared foods with 11 items;
4. Behaviors related to pre-prepared foods with five items.

The sociodemographic characteristics section included basic information such as gender, age, ethnicity, marital status, major, and education level of the participants. It also collected economic data like family monthly income and living expenses, as well as information regarding satisfaction with school dining services and personal weight satisfaction.

The knowledge section about pre-prepared foods contained three response options (correct, incorrect, and unsure), covering the definition of pre-prepared foods, food storage methods, current status, and nutritional value. Following the studies by Al Banna [21], questions 1, 2, 3, 4, 6, and 8 awarded 1 point for a “correct” response and 0 points for “incorrect” or “unsure” responses. For questions 5 and 7, an “incorrect” response was awarded 1 point, while “correct” or “unsure” responses were scored 0 points. Each participant’s total score ranged from 0 to 8. A score of 0–2 indicates a low level of knowledge, a score of 3–5 indicates a medium level of knowledge, and a score of 6–8 indicates a high level of knowledge.

The attitudes section included three response options (agree, disagree, and unsure) and addressed convenience, substitutability, hygiene standards, and preparation standards. Each “agree” response scored 1 point, while “disagree” or “unsure” responses scored 0 points. The total score for this section ranged from 0 to 11. A score of 0–3 indicates a low level of attitude, a score of 4–7 indicates a medium level of attitude, and a score of 8–11 indicates a high level of attitude.

The practices section focused on the frequency of purchasing and consuming pre-prepared foods and preparation of pre-prepared foods. This section used a 5-point Likert scale, scoring from 0 (“never”) to 4 (“always”), with a total score ranging from 0 to 20. A score of 0–6 indicates a low level of practice, 7–13 indicates a medium level of practice, and a score of 14–20 indicates a high level of practice.

Participants’ total scores ranged from 0 to 39, with a score of 0 to 13 indicating a low KAP level, a score of 14 to 26 indicating a medium KAP level, and a score of 15 to 39 indicating a high KAP level.

### 2.5. Validity and Reliability of the Questionnaire

We used Cronbach’s alpha to analyze reliability and test the internal consistency of the questionnaire. The results indicated that the internal consistency (Cronbach’s alpha, α) for the sections on knowledge, attitudes, and practices related to pre-prepared foods were 0.70, 0.71, and 0.72, respectively, with an overall internal consistency of 0.71, indicating acceptable reliability. 

Confirmatory factor analysis (CFA) was employed to validate the questionnaire’s validity. Using AMOS, a confirmatory factor analysis model was constructed and analyzed. The results indicated that the values of CMIN/df, RMR, RMSEA, and GFI were 4.875, 0.013, 0.050, and 0.932, respectively, all within acceptable ranges. This suggested good structural validity of the questionnaire.

### 2.6. Statistical Analyses

Statistical analyses were conducted using SPSS 26.0 software. Categorical data were described using frequencies and percentages. Normally distributed continuous data were described using means ± standard deviations, and non-normally distributed continuous data were described using medians and interquartile ranges. When data follow a skewed distribution, non-parametric analysis is the most appropriate data analysis method. Data were analyzed using Kruskal–Wallis tests or Wilcoxon rank-sum tests for univariate analysis. A multiple linear regression model was employed with knowledge, attitudes, and practices scores as dependent variables to identify factors associated with the scores on pre-prepared food knowledge, attitudes, and practices. The independent variables included were those found to be statistically significant in univariate analyses. A *p*-value of < 0.05 was considered statistically significant.

## 3. Results

### 3.1. Sociodemographic and Dietary Characteristics

This study, which received 1566 valid responses from a total of 1676 distributed questionnaires, revealed several key findings. The average age of the respondents was 22.24 ± 3.116 years, with a higher proportion of female participants (60%). The majority of participants were Han Chinese, and urban residents constituted 75.3% of the total sample. Unmarried participants made up 96.9% of the sample, 62.8% were undergraduates, and 5% were associate degree students. Most participants’ family incomes ranged from CNY 5000 to 20,000 per month, and over half of the participants had a monthly living allowance between CNY 1000 and 2000. Regarding satisfaction with school dining services, 50.3% of the participants rated it as average, while 9.8% were dissatisfied or very dissatisfied. Nearly half of the participants perceived themselves as overweight or very overweight, and 45.1% were attempting to lose weight. Calculations of participants’ BMI indicated that 20.6% were classified as overweight (Table 1).

### 3.2. Scores on Knowledge, Attitudes, and Practices Regarding Pre-Prepared Foods

The questionnaire items and scores are summarized in Table 2, Table 3 and Table 4, and the respondents’ degrees of prepared food knowledge, attitudes, and practices are summarized in Table 5. The overall mean score of the participants was 11.98 (0.00–32.00); of these, 66.09% had lower scores, indicating generally low overall scores. The mean score for knowledge related to pre-prepared foods was 5.42 (0.00–8.00), with 56.7% scoring well (score ≥ 6.00), indicating a high level of knowledge among participants. Most participants were well informed about the definition of pre-prepared foods (85.8%), the status of pre-prepared foods in take-out (82.9%), and whether pre-prepared foods contain preservatives and colorants (87.0%). However, they had lower knowledge regarding pre-prepared foods’ nutritional content and standards. The mean score for attitudes towards pre-prepared foods was 2.94 (0.00–11.00), with 66.028% scoring low, indicating a generally negative attitude. Most participants found pre-prepared foods convenient (73.6%) and considered them an inevitable outcome of a fast-paced era (61.4%). However, 86.0% of participants were concerned about using pre-prepared foods in school cafeterias, 84.5% believed that pre-prepared foods were not fresh, and 87.2% thought they could not replace daily meals. The mean score for behaviors related to pre-prepared foods was 3.62 (0.00–20.00), with 86.27% scoring low. Most participants and their families rarely purchased pre-prepared foods and did not encourage their use among peers. The frequency of participants or their families preparing pre-prepared foods was also low, indicating a low acceptance of pre-prepared foods.

### 3.3. Factors Influencing Knowledge, Attitudes, and Practices Regarding Pre-Prepared Foods

This study found that the status of being an only child, current education level, satisfaction with school dining services, and current attempts to manage weight significantly influenced the total scores of KAP regarding pre-prepared foods among university students (Table 6). These factors were further analyzed in a multiple linear regression (Table 7). The results showed that participants who were not only children had lower total scores compared to only children (β = −0.431, *p* < 0.05); participants with varying levels of satisfaction (satisfied, average, dissatisfied, very dissatisfied) with school dining services had lower total scores compared to those very satisfied (β = −1.385, −1.978, −2.100, −2.506, *p* < 0.01); participants with a master’s degree or higher had higher total scores compared to associate degree participants (β = 1.106, *p* < 0.05); and participants attempting to gain weight had lower total scores compared to those trying to lose weight (β = −0.737, *p* < 0.05).

Ethnicity, place of residence, only child status, current education level, family monthly income, monthly living expenses, and satisfaction with school dining services were significantly correlated with knowledge scores regarding pre-prepared foods (*p* < 0.05, Table 6). Multiple linear regression analysis incorporating these factors revealed that minority participants scored lower in knowledge than Han participants (β = −0.444, *p* < 0.01); non-only children scored lower than only children (β = −0.199, *p* < 0.05); undergraduate and master’s degree participants scored higher than associate degree participants (β = 0.965, *p* < 0.05; β = 1.252, *p* < 0.05); and participants with monthly living expenses between CNY 1000 and 2000 and between CNY 2000 and 5000 scored higher than those with less than CNY 1000 (β = 0.424, *p* < 0.05; β = 0.656, *p* < 0.01) (Table 7).

Gender, place of residence, only child status, satisfaction with school dining services, and BMI significantly connected with attitudes towards pre-prepared foods (*p* < 0.05) (Table 6). Multiple linear regression analysis showed that female participants had significantly lower attitude scores than male participants (β = −0.429, *p* < 0.01); participants with varying levels of satisfaction (satisfied, average, dissatisfied, very dissatisfied) with school dining services had lower attitude scores compared to those very satisfied (β = −1.142, −1.475, −2.015, −1.818, *p* < 0.01) (Table 7).

Gender, knowledge of pre-prepared foods, and attitudes towards pre-prepared foods all significantly influenced college students’ practices towards pre-prepared foods (β = 0.588, −0.127,0.324, *p* < 0.01) (Table 7).

## 4. Discussion

This study builds upon the literature documenting the knowledge, attitudes, and practices related to pre-prepared foods among university students in Central China. Overall, university students’ knowledge level about pre-prepared foods is relatively high, while overall KAP levels are generally low.

While most participants in our study demonstrated a good understanding of the definition of pre-prepared foods, the current situation of pre-prepared foods in take-out food, and whether additives are used in these meals, there are significant gaps in their knowledge. Particularly, their understanding of the nutritional components of pre-prepared foods and the related standards and systems is relatively lacking. These gaps in knowledge are crucial, as they highlight areas where educational and policy interventions can be targeted to improve the overall KAP levels [4]. According to iiMedia Research data, in 2023, 42.19% of Chinese netizens chose to know very well about prefabricated dishes, 54.74% chose to know relatively well, and 3.07% chose not to know at all [22]. As of December 2020, students accounted for the largest proportion of China’s netizens, accounting for 21.0% of the population [23]. Therefore, this may be one of the essential reasons for the low knowledge level of college students about pre-prepared foods. At the same time, it is worth noting that having a relatively high level of knowledge about prepared dishes does not mean that this will translate into actual behavior; for example, college students may recognize that consuming prepared dishes is not as healthy as it could be, but for practical reasons, they choose to consume prepared dishes for economy, speed, and convenience [24].

The overall KAP level, as well as the attitudes and practices of university students concerning pre-prepared foods, is generally low, which may align with China’s traditional food culture and could also be influenced by cultural, demographic, and social environmental factors. In recent years, as the pace of urban life has accelerated, pre-prepared foods, including fast food and meal kits, have emerged as a new highlight in the culinary world due to their convenience and speed [25]. However, since pre-prepared foods emerged as a topic of public interest, their existence and safety have been highly controversial. The Chinese have historically placed great importance on food. Moreover, Chinese cooking techniques are rich, intricate, and sophisticated, pursuing the ultimate in flavor, whereas pre-prepared foods, standardized and mass-produced, still fall short in replicating the taste of traditional cuisine [26,27,28]. Research indicates that the primary consumers of pre-prepared foods in China are middle-aged and young adults who face more significant pressures from both family and work and thus prefer pre-prepared foods as a means to simplify cooking [25]. However, with more time and unique campus dining environments and habits, university students may depend less on pre-prepared foods.

Research reports suggest that consumption of convenience food is more prominent among men [29,30,31] and those with lower educational backgrounds [31,32]. In our study, educational level was a factor influencing the knowledge scores about pre-prepared foods. Research by Kim et al. shows that education level affects consumers’ choices of Home Meal Replacements (HMRs) [1], and findings by Daniels et al. indicate that less-educated individuals tend to cook for themselves [33], consistent with our findings. Therefore, in future efforts to promote knowledge about pre-prepared foods, we can target populations with lower educational backgrounds. Additionally, we found that female participants scored lower than males in their attitudes towards pre-prepared foods. However, their behavior scores were higher than those of male participants, with no significant statistical difference in knowledge scores. This diverges from previous research findings. Boek et al. found that gender is an essential determinant in food choices among university students, with male students prioritizing price over nutritional value [34]. Similarly, research has shown that men prioritize convenience in food choices [33,35], yet we found that female university students are more likely to purchase or consume pre-prepared foods. Female students generally pay more attention to health and nutrition and are more sensitive to unhealthy ingredients in pre-prepared foods, such as high salt, high fat, and additives [36], which could be one reason for their more negative attitudes towards pre-prepared foods. However, food choices in real life are influenced by many factors, including social environment, taste preferences, and the flavor and texture of the food [25]. Hence, the reasons for higher behavior scores among female university students are multifaceted.

We discovered that participants who were very satisfied with school catering services scored higher in attitudes and overall KAP scores related to pre-prepared foods than other participants. Despite the rapid development of the ready-to-eat meal industry in China, anticipated to be a “trillion-yuan industry” [4], and gradually entering the back kitchens of campus catering due to its quick and convenient characteristics, there is still significant controversy over this trend. Many people are concerned about the nutritional content, freshness, hygiene, and food safety of pre-prepared foods used in school cafeterias and hold negative attitudes towards using pre-prepared foods in school dining services [37].

In our results, participants attempting to lose weight scored higher in KAP related to pre-prepared foods than those attempting to gain weight. Those trying to lose weight may be more inclined to purchase and consume low-calorie meals for fat reduction, while the variety and types of such meals offered in campus cafeterias are limited. University students primarily acquire light meals on campus through online purchases of bagged chicken breasts, buckwheat noodles, sandwiches, or take-out [38]. With the entry of pre-prepared foods into campus, vegetable salads and other ready-to-eat options in convenience stores also provide an alternative for the dieting population. Thus, students attempting to lose weight have a higher level of awareness and are more likely to purchase and consume pre-prepared foods. Moreover, participants with monthly living expenses between 1000 and 2000 yuan and those between 2000 and 5000 yuan scored higher in the knowledge dimension than those with less than 1000 yuan. On the one hand, living expenses are a significant source of income for university students, who rely predominantly on their monthly allowance for their day-to-day expenses, making it crucial for students to plan their monthly budget wisely. On the other hand, as an emerging product, the ready-to-eat meal industry still faces several issues, such as a lack of standardized industry norms [3], and the price of the same dish can vary significantly between brands. Therefore, students with lower living expenses are less likely to consider pre-prepared foods in their daily dining options, and their understanding of these meals may be lower than those with higher expenses.

According to research by Alzghoul et al., higher knowledge and attitude levels in the KAP model indicate better practice [39]. Interestingly, in our study, participants’ attitude scores were positively associated with their pre-prepared foods practice scores, but their knowledge scores were not, which differs from previous findings. The findings of Tofik Mohammed et al. suggest that the higher the level of knowledge about patient safety, the higher the level of practice [40]. The findings of Loofbourrow et al. similarly suggest that good knowledge and attitudes represent better practices [41]. The reasons for this discrepancy need to be further explored, and our guesses in that the pre-prepared foods in the Chinese market have not yet entered a large-scale and standardized development stage with multiple industrial attributes [42], although college students have a good understanding of pre-prepared foods, there may still be concerns regarding their purchase and consumption. This calls for further exploration of the psychology behind Chinese college students’ consumption of prefabricated dishes.

This study, the first of its kind, delves into the realm of college students’ knowledge, attitudes, and practices regarding pre-prepared foods. However, it is important to acknowledge the inadvertent limitations of our study. Firstly, the use of convenience sampling may have introduced bias that could not be fully addressed. Additionally, to ensure a higher response rate, the number of questions was kept limited, which may have hindered a comprehensive understanding of the knowledge, attitudes, and practices related to pre-prepared foods. Thirdly, the data were based on self-reported responses from participants, which could not be independently verified. Fourthly, as this is a cross-sectional study, establishing a cause-and-effect relationship is difficult. Fifthly, we did not explore the psychological factors influencing college students’ consumption of pre-prepared foods. Finally, the sample was limited to college students in central China, so the findings may not be generalizable to the broader population of China.

## 5. Conclusions

Understanding college students’ knowledge, attitudes, and practices regarding pre-prepared foods is key to promoting healthy eating among this group and advancing the development of the pre-prepared foods market in China. The future of pre-prepared foods looks promising, as it meets the diverse needs of various consumer groups and benefits from policy support. However, the industry faces significant challenges. It requires a unified standard system and standardized operational procedures, while issues like quality concerns, poor hygiene, and incomplete nutrition persist in some products. Although college students are not yet the primary consumer group in China, their university years mark a crucial transition to adulthood, potentially influencing their future habits. Therefore, alongside setting standards and enhancing oversight, consumers need to stay vigilant and make informed choices to ensure their health and nutrition. Future research should explore the social and psychological factors shaping college students’ knowledge, attitudes, and practices regarding pre-prepared foods. This will provide more evidence to support healthier eating choices for students and further the development of the pre-prepared foods industry.

## Figures and Tables

**Table 1:** Sociodemographic and dietary characteristics of study participants (N = 1566).

Variables and Category	Frequency	Percent
Gender		
Male	627	40.0
Female	939	60.0
Nationality		
The Han nationality	1249	79.8
Other	317	20.2
Place of residence		
Town	1179	75.3
Rural or suburban area	387	24.7
Whether you are an only child		
Yes	596	38.1
No	970	61.9
Marital status		
Unmarried	1518	96.9
Married or cohabiting	36	2.3
Divorced or separated	2	0.1
Widowed	3	0.2
Unknown	7	0.4
Education level		
College	79	5.0
Bachelor’s degree	984	62.8
Master’s degree or above	503	32.1
Grade level		
First grade	406	25.9
Second grade	344	22.0
Third grade	402	25.7
Fourth grade	282	18.0
Fifth grade and above	132	8.4
Monthly family income (RMB)		
Below 2000 yuan	44	2.8
2000 yuan to 5000 yuan	229	14.6
5000 yuan to 10,000 yuan	550	35.1
10,000 yuan to 20,000 yuan	488	31.2
20,000 yuan or above	255	16.3
Monthly living expenses		
Below 1000 yuan	108	6.9
1000 yuan to 2000 yuan	909	58.0
2000 yuan to 5000 yuan	517	33.0
Above 5000 yuan	32	2.0
Overall level of satisfaction with school meals		
Very satisfied	80	5.1
Satisfied	545	34.8
Fair	787	50.3
Dissatisfied	116	7.4
Very dissatisfied	38	2.4
How to perceive your weight		
Too thin	56	3.6
Somewhat thin	194	12.4
Just right	566	36.1
Somewhat fat	646	41.3
Too fat	104	6.6
Currently trying to figure out how to manage your weight		
Lose weight	707	45.1
Maintain current weight	698	44.6
Weight gain	161	10.3
BMI		
Thin	232	15.0
Normal	998	64.4
Overweight	319	20.6

**Table 2:** Summary of questions and responses for assessment of pre-prepared foods knowledge of Chinese university students (N = 1566).

Statements	Responses, *n* (%)			The Average Score of the Question
	True	False	Don’t Know	
Prepared food refers to finished or semi-finished products made from agricultural, animal, poultry and aquatic products as raw materials, with various auxiliary materials, after pre-processing.	1344 (85.8)	42 (2.7)	180 (11.5)	0.86 ± 0.35
Reheating food may cause food contamination.	1101 (70.3)	215 (13.7)	250 (16.0)	0.70 ± 0.46
A large proportion of take-away food is prepared dishes.	1298 (82.9)	40 (2.6)	228 (14.6)	0.83 ± 0.38
Prepared vegetables are generally high in carbohydrates, protein, and fat.	759 (48.5)	346 (22.1)	461 (29.4)	0.48 ± 0.50
Prepared vegetables are generally high in vitamins, minerals, and biologically active ingredients.	244 (15.6)	837 (53.4)	485 (31.0)	0.53 ± 0.50
Long-term consumption of prepared vegetables may lead to excessive salt intake and increase the risk of high blood pressure.	1231 (78.6)	54 (3.4)	281 (17.9)	0.79 ± 0.41
At present, there is a unified standard system, certification system and traceability system for prepared vegetables.	379 (24.2)	561 (35.8)	626 (40.0)	0.36 ± 0.48
Preservatives, additives, coloring, etc. may be added to pre-prepared vegetables for long-term preservation and to ensure good taste.	1362 (87.0)	44 (2.8)	160 (10.2)	0.87 ± 0.34

**Table 3:** Summary of questions and responses for assessment of pre-prepared foods attitudes of Chinese university students (N = 1566).

Statements	Responses, *n* (%)			Average Score
	Agree	Disagree	Don’t Know	
I think that prepared food has brought convenience to our eating and drinking.	1152 (73.6)	308 (19.7)	106 (6.8)	0.74 ± 0.44
I do not mind that the takeaway food is prepared food of the heated ready-to-eat type.	459 (29.3)	999 (63.8)	108 (6.9)	0.29 ± 0.46
I do not mind that the school cafeteria uses prepared food to prepare three meals a day.	159 (10.2)	1346 (86.0)	61 (3.9)	0.10 ± 0.30
I think the preparation of prepared food is technically demanding.	593 (37.9)	685 (43.7)	288 (18.4)	0.38 ± 0.49
I think the prepared food is clean and hygienic.	201 (12.8)	985 (62.9)	380 (24.3)	0.13 ± 0.36
I have a positive attitude towards the development of the prepared food industry.	487 (31.1)	727 (46.4)	352 (22.5)	0.31 ± 0.46
I do not mind using prepared food at home to prepare three meals a day.	258 (16.5)	1204 (76.9)	104 (6.6)	0.16 ± 0.37
I think prepared food is the inevitable result of the fast-paced era.	961 (61.4)	444 (28.4)	161 (10.3)	0.61 ± 0.49
I think the freshness of prepared food is higher.	91 (5.8)	1323 (84.5)	152 (9.7)	0.06 ± 0.23
I think that prepared food can replace daily meals in the future.	99 (6.3)	1366 (87.2)	101 (6.4)	0.06 ± 0.24
I think it is safe and healthy for the elderly and teenagers to consume prepared food.	138 (8.8)	1207 (77.1)	221 (14.4)	0.09 ± 0.28

**Table 4:** Summary of questions and responses for assessment of pre-prepared foods practices of Chinese university students (N = 1566).

Statements	Responses, *n* (%)					Average Score
	Never	Rarely	Sometimes	Often	Always	
Do you often buy and consume prepared food?	268 (17.1)	687 (43.9)	550 (35.1)	54 (3.4)	7 (0.4)	1.26 ± 0.80
Do you encourage people around you to eat prepared food?	928 (59.3)	461 (29.4)	134 (8.6)	30 (1.9)	13 (0.8)	0.56 ± 0.80
Do you make your own prepared food?	984 (62.8)	372 (23.8)	174 (11.1)	26 (1.7)	10 (0.6)	0.54 ± 0.80
Do your family members often buy and eat prepared food?	747 (47.7)	585 (37.4)	218 (13.9)	12 (0.8)	4(0.3)	0.69 ± 0.76
Does your family make their own prepared food?	898 (57.3)	455 (29.1)	190 (12.1)	17 (1.1)	6(0.4)	0.58 ± 0.77

**Table 5:** The scores and degrees of pre-prepared food knowledge, attitudes, and practices of the respondents (N = 1566).

Scores	Frequency	Percent	Degree	Frequency	Percent
Knowledge score					
0.00	22	1.4	bad	112	7.2
1.00	36	3.0			
2.00	54	3.4			
3.00	120	7.7	medium	566	36.1
4.00	201	12.8			
5.00	245	15.6			
6.00	408	26.1	good	888	56.7
7.00	328	20.9			
8.00	152	9.7			
Attitude score					
0.00	174	11.1	bad	1034	66.0
1.00	261	16.7			
2.00	309	19.7			
3.00	290	18.5			
4.00	226	14.4	medium	469	30.0
5.00	125	8.0			
6.00	83	5.3			
7.00	35	2.2			
8.00	23	1.5	good	63	4.0
9.00	20	1.3			
10.00	7	0.4			
11.00	13	0.8			
Practice score					
0.00	165	10.5	bad	1351	86.3
1.00	217	13.9			
2.00	223	14.2			
3.00	214	13.7			
4.00	234	14.9			
5.00	182	11.6			
6.00	116	7.4			
7.00	88	5.6	medium	209	13.4
8.00	49	3.1			
9.00	30	1.9			
10.00	29	1.8			
11.00	6	0.4			
12.00	5	0.3			
13.00	2	0.1			
15.00	3	0.2	good	6	0.4
17.00	1	0.1			
20.00	2	0.1			

**Table 6:** Respondents’ pre-prepared foods knowledge, attitudes, and practices by their sociodemographic and dietary characteristics (N = 1566).

Characteristics	Knowledge Score		Attitude Score		Practice Score		Total Score	
	Mean ± SD	*p* Value	Mean ± SD	*p* Value	Mean ± SD	*p* Value	Mean ± SD	*p* Value
Gender			<					
Male	5.34 ± 1.87	0.23	3.21 ± 2.35	<0.01	3.37 ± 2.74	<0.01	11.92 ± 4.43	0.55
Female	5.48 ± 1.76		2.76 ± 2.04		3.79 ± 2.64		12.02 ± 4.18	
Nationality								
Han	5.54 ± 1.75	<0.01	2.86 ± 2.04	0.15	3.52 ± 2.54	0.05	11.92 ± 4.02	0.58
Other	4.95 ± 1.95		3.24 ± 2.62		4.03 ± 3.19		12.23 ± 4.76	
Place of residence								
Town	5.49 ± 1.78	0.02	3.00 ± 2.15	0.02	3.58 ± 2.67	0.30	12.06 ± 4.13	0.28
Rural or suburban area	5.23 ± 1.87		2.75 ± 2.56		3.74 ± 2.75		11.73 ± 4.31	
Whether you are an only child								
Yes	5.65 ± 1.68	<0.01	3.08 ± 2.13	0.01	3.53 ± 2.45	0.73	12.26 ± 3.84	0.03
Not	5.28 ± 1.86		2.85 ± 2.20		3.68 ± 2.82		11.81 ± 4.37	
Marital status								
Unmarried	5.42 ± 1.80	0.75	2.95 ± 2.16	0.16	3.62 ± 2.66	0.85	11.99 ± 4.17	0.51
Married or cohabiting	5.53 ± 1.50		2.81 ± 2.56		3.56 ± 2.72		11.89 ± 4.39	
Divorced or separated	3.00 ± 4.24		1.00 ± 1.41		10.50 ± 13.44		14.50 ± 7.78	
Widowed	6.33 ± 0.58		4.00 ± 5.29		3.00 ± 3.61		13.33 ± 2.52	
Unknown	5.29 ± 2.36		1.57 ± 2.15		2.71 ± 2.21		9.57 ± 4.72	
Education level								
College	4.22 ± 2.02	<0.01	3.32 ± 2.75	0.70	3.84 ± 3.33	0.59	11.37 ± 4.77	0.02
Bachelor’s degree	5.38 ± 1.83		2.91 ± 2.18		3.55 ± 2.61		11.84 ± 4.16	
Master’s degree or above	5.71 ± 1.64		2.92 ± 2.07		3.72 ± 2.72		12.35 ± 4.09	
Grade level								
First grade	5.24 ± 1.83	0.12	2.83 ± 2.21	0.08	3.59 ± 2.86	0.08	11.66 ± 4.53	0.07
Second grade	5.55 ± 1.75		2.87 ± 2.18		3.47 ± 2.44		11.88 ± 3.92	
Third grade	5.52 ± 1.79		3.10 ± 2.05		3.74 ± 2.53		12.36 ± 3.89	
Fourth grade	5.40 ± 1.86		2.99 ± 2.19		3.41 ± 2.66		11.79 ± 4.01	
Fifth grade and above	5.42 ± 1.78		2.83 ± 2.40		4.19 ± 3.20		12.45 ± 4.78	
Monthly family income (RMB)								
Below 2000 yuan	4.70 ± 2.22	<0.01	2.59 ± 2.46	0.15	3.82 ± 3.72	0.15	11.11 ± 4.78	0.25
2000 yuan to 5000 yuan	5.14 ± 1.98		3.00 ± 2.45		3.82 ± 2.77		11.96 ± 4.49	
5000 yuan to 10,000 yuan	5.33 ± 1.83		2.90 ± 2.14		3.76 ± 2.63		11.99 ± 4.26	
10,000 yuan to 20,000 yuan	5.59 ± 1.68		2.82 ± 1.96		3.38 ± 2.47		11.78 ± 3.74	
20,000 yuan or above	5.70 ± 1.65		3.24 ± 2.32		3.57 ± 2.91		12.51 ± 4.37	
Monthly living expenses								
Below 1000 yuan	4.69 ± 2.23	<0.01	2.78 ± 2.49	0.30	3.96 ± 3.70	0.23	111.43 ± 4.87	0.41
1000 yuan to 2000 yuan	5.34 ± 1.81		2.95 ± 2.21		3.69 ± 2.58		11.98 ± 4.25	
2000 yuan to 5000 yuan	5.70 ± 1.67		2.89 ± 1.96		3.44 ± 2.61		12.03 ± 3.84	
Above 5000 yuan	5.84 ± 1.37		3.91 ± 3.23		3.31 ± 2.67		13.06 ± 4.76	
Overall level of satisfaction with school meals								
Very satisfied	4.98 ± 1.86	0.01	4.19 ± 2.95	<0.01	4.43 ± 3.81	0.07	13.59 ± 5.08	<0.01
Satisfied	5.40 ± 1.80		3.14 ± 2.21		3.77 ± 2.65		12.32 ± 4.12	
Fair	5.43 ± 1.78		2.79 ± 2.02		3.46 ± 2.51		11.68 ± 4.05	
Dissatisfied	5.82 ± 1.85		2.26 ± 1.80		3.53 ± 2.80		11.60 ± 4.05	
Very dissatisfied	5.37 ± 1.99		2.42 ± 2.60		3.32 ± 3.23		11.11 ± 5.21	
How to perceive your weight								
Too thin	5.27 ± 2.02	0.12	3.13 ± 2.48	0.16	3.14 ± 2.49	0.49	11.54 ± 4.05	0.78
Somewhat thin	5.46 ± 1.75		2.84 ± 2.16		3.55 ± 2.56		11.85 ± 4.19	
Just right	5.33 ± 1.77		2.91 ± 2.18		3.65 ± 2.69		11.90 ± 4.06	
Somewhat fat	5.56 ± 1.74		2.91 ± 2.16		3.68 ± 2.68		12.15 ± 4.27	
Too fat	5.07 ± 2.26		3.33 ± 2.12		3.45 ± 3.04		11.85 ± 4.27	
Currently trying to figure out how to manage your weight								
Lose weight	5.47 ± 1.79	0.65	3.03 ± 2.22	0.40	3.76 ± 2.78	0.06	12.25 ± 4.37	0.08
Maintain current weight	5.40 ± 1.78		2.84 ± 2.10		3.59 ± 2.64		11.83 ± 3.94	
Weight gain	5.32 ± 1.96		2.93 ± 2.27		3.17 ± 2.39		11.42 ± 4.26	
BMI								
Thin	5.34 ± 1.93	0.96	2.72 ± 2.00	0.03	3.84 ± 2.70	0.23	11.91 ± 3.94	0.68
Normal	5.46 ± 1.75		2.90 ± 2.17		3.58 ± 2.72		11.93 ± 4.17	
Overweight	5.41 ± 1.87		3.23 ± 2.29		3.58 ± 2.58		12.22 ± 4.40	

**Table 7:** Multiple linear regression models identifying the factors associated with pre-prepared foods knowledge, attitudes, and practices among study participants (N = 1566).

Model	Variable	β	95% CI	*p* Value
Model 1 Knowledge scores	(const)	3.745	3.011, 4.478	<0.01
	Nationality			
	The Han nationality	RC		
	Other	−0.444	−0.669, −0.220	<0.01
	Place of residence			
	Town	RC		
	Rural or suburban area	0.062	−0.166, 0.290	0.59
	Whether you are an only child			
	Yes	RC		
	No	−0.199	−0.390, −0.007	0.04
	Education level			
	College	RC		
	Bachelor’s degree	0.965	0.550, 1.380	<0.01
	Master’s degree or above	1.252	0.823, 1.681	<0.01
	Monthly family income (RMB)			
	Below 2000 yuan	RC		
	2000 yuan to 5000 yuan	0.188	−0.401, 0.777	0.53
	5000 yuan to 10,000 yuan	0.084	−0.507, 0.674	0.78
	10,000 yuan to 20,000 yuan	0.260	−0.345, 0.866	0.40
	20,000 yuan or above	0.214	−0.426, 0.854	0.51
	Monthly living expenses			
	Below 1000 yuan	RC		
	1000 yuan to 2000 yuan	0.424	0.032, 0.817	0.03
	2000 yuan to 5000 yuan	0.656	0.214, 1.097	<0.01
	Above 5000 yuan	0.643	−0.119, 1.406	0.10
	Overall level of satisfaction with school meals			
	Very satisfied	RC		
	Satisfied	0.193	−0.223, 0.610	0.36
	Fair	0.216	−0.193, 0.625	0.30
	Dissatisfied	0.507	0, 1.015	0.05
	Very dissatisfied	0.240	−0.443, 0.922	0.49
Model 2 Attitudes scores	(const)	4.700	4.184, 5.216	<0.01
	Gender			
	Male	RC		
	Female	−0.429	−0.647, −0.212	<0.01
	Place of residence			
	Town	RC		
	Rural or suburban area	−0.210	−0.468, 0.048	0.11
	Whether you are an only child			
	Yes	RC		
	No	−0.182	−0.41, 0.047	0.12
	Overall level of satisfaction with school meals			
	Very satisfied	RC		
	Satisfied	−1.142	−1.652, −0.632	<0.01
	Fair	−1.475	−1.975, −0.976	<0.01
	Dissatisfied	−2.015	−2.631, −1.400	<0.01
	Very dissatisfied	−1.818	−2.655, −0.981	<0.01
	BMI	0.000	0, 0	0.14
Model 3 Practices scores	(const)	3.004	2.537,3.471	<0.01
	Knowledge scores	−0.127	−0.198,−0.056	<0.01
	Attitudes scores	0.324	0.264,0.383	<0.01
	Gender			
	Male	RC		
	Female	0.588	0.326,0.851	<0.01
Model 4 Total scores	(const)	13.471	12.186, 14.757	<0.01
	Whether you are an only child			
	Yes	RC		
	No	−0.431	−0.855, −0.007	0.05
	Overall level of satisfaction with school meals			
	Very satisfied	RC		
	Satisfied	−1.385	−2.361, −0.409	<0.01
	Fair	−1.978	−2.935, −1.021	<0.01
	Dissatisfied	−2.100	−3.285, 0.916	<0.01
	Very dissatisfied	−2.506	−4.109, −0.903	<0.01
	Education level			
	College	RC		
	Bachelor’s degree	0.593	−0.363, 1.549	0.22
	Master’s degree or above	1.106	0.116, 2.096	0.03
	Currently trying to figure out how to manage your weight			
	Lose weight	RC		
	Maintain current weight	−0.414	−0.848, 0.021	0.06
	Weight gain	−0.737	−1.448, −0.027	0.04

Note: β = regression coefficient; RC = reference category; CI = confidence interval.

## Data Availability

The datasets used in the present study are available from the corresponding authors on reasonable request.
